# Self-face recognition under self-implicating threat: preserved self-prioritization and recalibrated control dynamics

**DOI:** 10.1186/s41235-026-00744-8

**Published:** 2026-07-01

**Authors:** ChaoZheng Huang, Hui Jiang, HanBin Sang, Pei Xie, AiBao Zhou

**Affiliations:** 1 School of Psychology, Northwest Normal University, Lanzhou, 730070 China; 2 School of Psychology, Sichuan Normal University, Chengdu, 610068 China; 3 Key Laboratory of Child Cognition and Behavior Development of Hainan Province, Qiongtai Normal University, Haikou, 571127 China

**Keywords:** Self-face, Neural oscillations, Criminal context, Self-processing

## Abstract

Self-related information is typically processed with high priority, but in everyday life self-relevant cues are often embedded in socially evaluative or self-related crime contexts. The present study examined whether a self-related crime context disrupts self-face prioritization or instead recalibrates its behavioral and neural expression. Participants completed a self/other face judgment task after three contextual framings: self-related crime, self-irrelated crime, and self-related non-crime. Event-related potentials and time–frequency activity were recorded to characterize the temporal dynamics of face processing. Behaviorally, self-faces were recognized faster than other-faces overall, but responses to self-faces were slower in the self-related crime condition than in the other contexts. The N170 was not modulated by face type or context, suggesting that early structural encoding was relatively insensitive to contextual framing. In contrast, self-faces elicited larger N250 and P3 amplitudes than other-faces across contexts, indicating preserved self-related identity activation and post-perceptual evaluation. Crime-related framing also increased N250 and P3 amplitudes more broadly, consistent with enhanced contextual salience or evaluative significance. Time–frequency analyses further showed context-dependent theta and alpha modulations, suggesting altered control dynamics and attentional gating when self-relevance was paired with crime-related meaning. These findings indicate that self-implicating negative contexts do not eliminate self-prioritization. Instead, they preserve core electrophysiological indices of self-processing while recalibrating behavioral efficiency and oscillatory control dynamics.

## Introduction

The prioritization of self-related information is a fundamental feature of human cognition. Self-relevant stimuli are often detected, categorized, and remembered more efficiently than stimuli associated with other people, suggesting that the self can function as a powerful organizing principle in perception, attention, memory, and decision-making (Cheng et al., 2022; Humphreys & Sui, 2016; Sui & Humphreys, 2015). This self-prioritization effect has been demonstrated across a range of paradigms, including self-face recognition, self-voice recognition, perceptual matching, and memory tasks (Candini et al., 2014; Cygan et al., 2014; Devue & Brédart, 2011).

Under neutral or low-threat conditions, self-related identity cues generally show processing advantages. Self-faces are typically processed more efficiently than familiar or unfamiliar faces (Devue & Brédart, 2011), and self-voices also show privileged processing in implicit and explicit recognition tasks (Candini et al., 2014). Neurophysiological and neuroimaging evidence further suggests that self-related identity processing is not reducible to familiarity alone. For example, self-faces elicit larger P3 amplitudes than familiar faces (Cygan et al., 2014) and preferentially recruit anterior regions related to self-referential and affective processing, including the anterior cingulate cortex and anterior insula. In contrast, familiar faces more strongly engage posterior cingulate regions associated with semantic and episodic memory (Chavez, 2021; Qin et al., 2012). These findings indicate that self-related identity cues are processed through mechanisms involving personal significance, attentional priority, and affective-evaluative meaning, rather than perceptual familiarity alone.

However, self-related processing does not occur in isolation. In everyday social life, self-identity is often embedded in evaluative, emotional, and morally meaningful contexts, such as situations involving responsibility, blame, wrongdoing, or social judgment. Contextual semantics can substantially influence face processing, particularly when stimuli are ambiguous or evaluatively significant (Rubianes et al., 2024; Wieser et al., 2014). Importantly, prior evidence shows that social threat can modulate the self-face advantage, suggesting that self-prioritization is not entirely fixed but can vary with the motivational meaning of the context (Ma & Han, 2009). Understanding how self-prioritized processing changes in such contexts is therefore important for clarifying both the robustness and the boundary conditions of self-processing advantages.

A crime-related context provides a particularly informative extension of, this issue because it is not merely a negative background. Rather, it may combine self-relevance with moral violation, perceived culpability, social evaluation, and the need for defensive regulation. Moral violation and perceived culpability are closely linked to self-conscious moral emotions, such as guilt and shame, which arise when one’s actions are evaluated against moral standards and their implications for the self (Tangney et al., 2007). Contexts involving possible negative evaluation of the self can also elicit social-evaluative threat and defensive self-regulation (Dickerson & Kemeny, 2004; Sherman & Cohen, 2006). Thus, crime-related self-involvement provides a useful context for testing whether self-prioritization remains stable or is constrained by the motivational meaning attached to the self.

At the same time, crime-related framing should not be equated with subjective self-threat a priori. Evaluatively salient contexts can amplify semantic interpretation and attentional engagement even without direct evidence of self-threat (Barrett et al., 2011; Wieser & Brosch, 2012). Crime-related framing may therefore influence face processing in two ways. First, it may operate as a general contextual signal that increases the salience of socially relevant stimuli. Second, when crime-related meaning is explicitly linked to the self, it may impose additional self-specific regulatory demands. This distinction is important for interpreting post-perceptual ERP components. If crime-related framing mainly reflects generalized contextual salience, N250 and P3 modulations may occur for both self- and other-faces. If self-related crime meaning additionally recruits defensive regulation, effects may be stronger for self-faces, especially at later evaluative stages or in overt behavior.

Face recognition is commonly conceptualized as a multistage process that progresses from structural encoding to identity recognition and semantic evaluation (Bruce & Young, 1986). Within this architecture, self-faces appear to follow partially distinct processing trajectories (Zhou et al., 2020). The N170 indexes early structural encoding and feature integration (Joyce & Rossion, 2005), and is generally face-sensitive but relatively insensitive to identity or higher-order contextual meaning in largely neutral paradigms (Gao et al., 2025; Wieser et al., 2014; Żochowska et al., 2023). Whether this relative invariance extends to crime-related framing remains unclear. Later ERP components may be more sensitive to identity and contextual meaning. The N250 reflects activation of stored identity representations and familiarity-based recognition (Schweinberger & Neumann, 2016), Self-faces can evoke N250 responses even when task-irrelevant or weakly attended, suggesting relatively automatic activation of self-identity representations (Zimmermann & Eimer, 2014). whereas the P3 is associated with semantic evaluation and attentional resource allocation (Polich, 2007; Zhu et al., 2024). Its amplitude increases when contextual cues are congruent with facial information (Tiberghien, 1986; Wieser et al., 2014), and self-faces generally elicit larger P300 responses than close others or neutral faces (Żochowska & Nowicka, 2024), indicating that self-prioritization may be especially pronounced at post-perceptual stages.

Neural oscillations provide a complementary index of these processing dynamics. Frontal midline theta (4–8 Hz) has often been linked to cognitive control and conflict monitoring (Cavanagh & Frank, 2014), whereas alpha suppression is commonly associated with increased attentional allocation or cortical disinhibition toward salient stimuli (Alzueta et al., 2020; Qin et al., 2009). In self-relevant negative contexts, theta activity may reflect changes in control recruitment, inhibitory gating, or defensive disengagement (Kotlewska et al., 2023), whereas alpha suppression may indicate heightened vigilance or increased attentional allocation (Codispoti et al., 2023).These components therefore provide useful indices for examining whether crime-related framing influences self-face processing primarily at identity-related and post-perceptual stages.

Two theoretical perspectives motivate the present study. The Implicit Positive Association account proposes that self-related stimuli are intrinsically associated with positive value, such that violations of positive self-associations may weaken the usual self-processing advantage (Ma & Han, 2009, 2010; McCrackin & Itier, 2018). In contrast, the Self-Attention Network model emphasizes prioritized allocation of attention to self-relevant information (Humphreys & Sui, 2016; Sui & Rotshtein, 2019). By placing self-face recognition in a crime-related context, the present study extends these models beyond neutral or generally evaluative settings and examines whether self-prioritization is modulated when self-identity is embedded in morally negative contextual meaning.

The focus on crime-related context also has broader relevance. In forensic and deception-related research, crime-relevant information has long been used to examine whether individuals recognize details that are specifically linked to an event, as in the guilty-knowledge and concealed-information traditions (Ben-Shakhar & Elaad, 2003; Lykken, 1959; Verschuere et al., 2011). Reaction-time research further suggests that latency measures can capture the additional cognitive demands associated with deception or concealment (Suchotzki et al., 2017). The present study uses a controlled crime-related context to address a more basic cognitive question: whether linking one’s identity to a morally negative event changes the processing of core self-identity cues. In this sense, the crime-related context is theoretically important for testing boundary conditions of self-processing models and practically relevant as an exploratory step toward understanding how self-event linkage may be reflected in behavioral and neural responses.

To test these possibilities, participants performed a self/other face judgment task in which each face was preceded by one of three contextual framings: self-related crime, in which the participant was framed as the perpetrator; self-irrelated crime, in which crime information was present but did not implicate the participant; and self-related non-crime, a neutral self-involving event. This design allowed us to distinguish self-relevance from crime-related contextual salience and examine whether linking one’s identity to criminal wrongdoing alters self-face processing in a self-specific manner or as part of broader contextual modulation (Deng et al., 2021; Gao et al., 2018; Na et al., 2021; Ünal et al., 2025). Thus, the simulated crime-related framing was not intended to directly mirror real criminal interrogation. Instead, it operationalized crime-related self-involvement under controlled laboratory conditions, allowing us to isolate whether crime relevance, self-relevance, or their combination modulates the processing of self-identity cues.

We expected an overall self-prioritization effect, with self-faces processed more efficiently than other-faces. Contextual framing was expected to exert its strongest influence at post-perceptual rather than early structural stages: N170 was expected to show limited contextual modulation, whereas N250 and P3 were expected to be more context-sensitive. If crime-related framing primarily increased general contextual salience, N250 and P3 modulations could occur for both self- and other-faces; if self-related crime additionally recruited self-specific regulation, effects could be stronger for self-faces, especially at later stages or in behavior. In the oscillatory domain, theta and alpha effects were treated as competing possibilities: frontal theta might increase with control recruitment, but might decrease if self-related crime framing promoted inhibitory gating or defensive disengagement; alpha suppression was considered a plausible marker of increased attentional allocation or vigilance. Thus, the study aimed to determine whether contextual influences on self-face recognition are better characterized as generalized contextual salience, self-specific defensive modulation, or their combination.

## Methods

### Participants

A priori power was calculated in G*Power 3.1.9.2 (Faul et al., 2007) for a within-subjects ANOVA. With an effect size of f = 0.25, an alpha level of 05, and statistical power (1 – β) set at .80, the minimum required sample size was 18 participants. Because EEG data are susceptible to trial loss caused by artifacts, and because stable ERP estimates require a sufficient number of artifact-free trials, we recruited more participants than the minimum required sample to maintain adequate statistical power after data exclusion. A total of 32 undergraduate students were recruited for the EEG experiment. Data from three participants were excluded because more than 25% of their averaged EEG trials contained artifacts, leaving 29 valid datasets for analysis (Mage = 22.48 years, SD = 2.46; 11 males, 18 females). This final sample size was also consistent with previous EEG studies on self-face recognition (Brunet, 2023; Zhong et al., 2016).

Prior to the experiment, all participants received a detailed explanation of the task procedure and were informed of their right to withdraw at any time. All participants were physically healthy, had no history of psychiatric or neurological disorders, were right-handed, and reported normal or corrected-to-normal vision. On the day of testing, all participants were in good mental condition and had no prior experience with similar experiments. The study was approved by the Ethics Committee of Northwest Normal University. Written informed consent was obtained from all participants, and they received monetary compensation upon completion of the experiment.

### Apparatus and materials

The experimental procedure was programmed using E-Prime 3.0. Visual stimuli were presented on a 23.8-inch Lenovo S24e-20 monitor (resolution: 1920 × 1080; refresh rate: 60 Hz), driven by an NVIDIA GTX 1050Ti graphics card. Participants provided responses via a standard keyboard. All tasks were conducted in a quiet behavioral laboratory, with participants seated approximately 60 cm from the screen.

Both text and image stimuli were generated and controlled using E-Prime 3.0. The experimental task required participants to respond to self-face and other-face stimuli. All stimuli were presented against a black background (RGB: 0, 0, 0). Face stimuli had a resolution of 511 × 591 pixels.

Participants should completed the offline simulated theft task to establish a first-person episodic memory of committing a theft or performing a neutral act. The task materials were printed on A4 paper and placed in sealed envelopes. Before the experiment, participants were told that three envelopes contained different tasks and were asked to draw one at random (in fact, all envelopes contained the same task). Following Deng et al. (2021), the task simulated a theft scenario. *Specifically, participants were instructed to deliver apples to Room 406 of the Special Education Building at Northwest Normal University. Upon entering the room, if asked, “What are you doing here?”, they were instructed to respond, “I am here to deliver apples.” They were further instructed to proceed to Room 445, locate the leftmost drawer of a metal cabinet, retrieve a yellow envelope containing 2000 RMB in cash, take the money, conceal it, return the envelope to the drawer, and then go back to the testing room without being detected.* If questioned (e.g., “What are you doing?”), participants were instructed to lie and say, “I am helping someone pick up an item.” If participants instead admitted, “I am doing an experiment,” the session would be terminated. In practice, all participants successfully completed the simulated theft task.

Face stimuli were prepared prior to the experiment. Self-face photographs were taken using a Logitech C922 PRO webcam, with participants positioned approximately 60 cm from the camera and instructed to maintain a neutral facial expression. For the other-face condition, ten unfamiliar face identities were used as other-face stimuli. These faces were matched to the self-face stimuli in terms of sex, age (± 2 years), luminance, contrast, and background. All images were converted to grayscale and normalized for mean luminance and standard deviation. To minimize the influence of extraneous facial features and ensure that participants focused on internal facial information, all face images were edited using Adobe Photoshop CS4 to remove hair, cropped into an oval shape, and centrally positioned.

### Procedure

Before the experiment, participants signed an informed consent form and provided demographic information, including name, gender, age, and handedness. In the formal session, each participant completed a face recognition task consisting of 420 trials. Within this task, participants were required to make judgments about self-faces across three contextual conditions: self-related crime, self-irrelated crime, and self-related non-crime, with 140 trials in each condition, There were 70 trials each for the self-face and the other-face. The task was divided into three blocks, with short breaks allowed between blocks. Both face stimuli and contextual information were presented in a randomized order. Before the formal task, participants completed 10 practice trials to familiarize themselves with the procedure. After each contextual priming block, participants were allowed to rest, and resumed the task by pressing a designated key. The entire experiment lasted approximately 90 min.

**Fig. 1:**
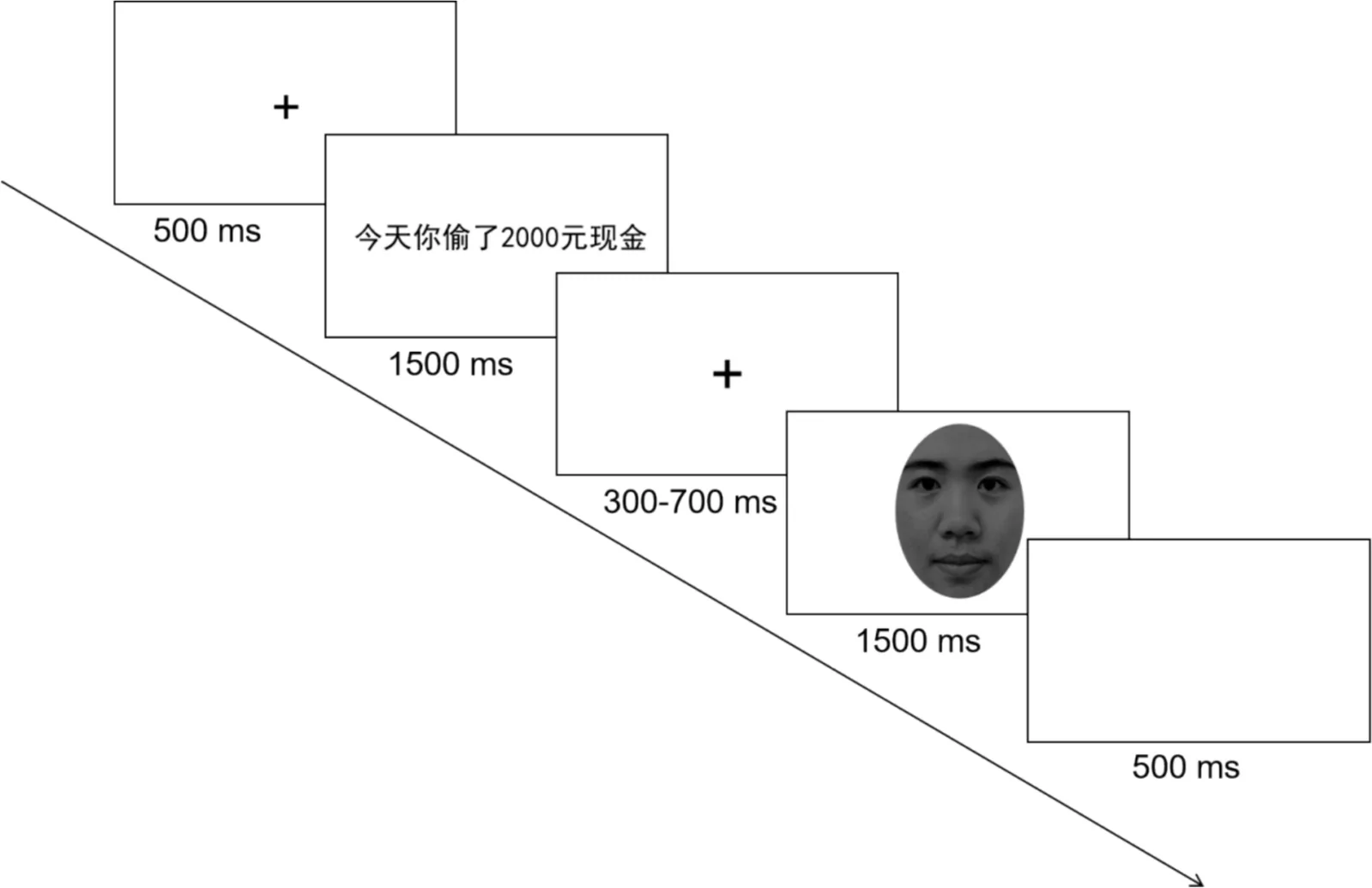
Schematic of the face recognition process.”今天你偷了2000元现金”means” Today, You stole 2000 yuan in cash”

Following the paradigm of Klein et al., (2015), contextual information was presented prior to the self-face recognition task to examine its influence on face processing. Specifically, each trial began with a fixation cross (“ + ”) displayed at the center of the screen for 500 ms to direct participants’ gaze. This was followed by a contextual statement, such as “Today you stole 2000 yuan in cash” (self-related crime information), “Today you stole confidential documents” (self-irrelated crime information), or “Today you delivered apples” (self-related non-crime information), presented for 1500 ms. Subsequently, another fixation cross appeared for 300–700 ms, after which either a self-face or an other-face stimulus was randomly presented for 1500 ms. Participants were instructed to judge, as quickly and accurately as possible, whether the face belonged to themselves. Responses were made using the index fingers of both hands, with the keys “F” (self-face) and “J” (non-self-face) counterbalanced across participants. Each trial concluded with a blank screen lasting 500 ms (see Fig. 1).

### EEG data recording and analysis

EEG was recorded using a 64-channel electrode cap (ANT Neuro, Enschede, Netherlands) arranged according to the extended international 10–20 system. During online recording, signals were referenced to CPz, and a sampling rate of 1000 Hz/channel. Electrode impedance was maintained below 10 kΩ (Zhang et al., 2024). EEG data were processed offline. Preprocessing was conducted in MATLAB using the EEGLAB toolbox (v2019.1). The main steps were: (1) re-referencing all electrodes to the M1/M2 average; (2) applying a 0.1-Hz high-pass and a 30-Hz low-pass filter; (3) performing independent component analysis (runica) and, based on expert inspection together with ICLabel, removing ocular and myogenic components with classification probability > 90% (Zhang et al., 2024); and (4) rejecting segments with absolute voltage exceeding ± 100 μV (Zhang et al., 2024). After artifact rejection and elimination of incorrect trials, more than 80% of valid trials were retained in each condition. Data epochs extended from − 200 ms to 800 ms relative to stimulus onset, with baseline correction applied to the − 200–0 ms window.

For time-domain analysis, ERPLAB in MATLAB was used. ERP components were analyzed within specific time windows based on prior studies (Klein et al., 2015; Schweinberger & Neumann, 2016; Zhong et al., 2016) and the aims of the current study. The mean amplitude of the N170 (120–180 ms) was measured at occipito-parietal sites (P7/8, PO7/8); the N250 (280–340 ms) at frontal (F3/z/4) and fronto-central (FC3/z/4) sites; and the P3 (390–490 ms) at central (C3/z/4), centro-parietal (CP3/z/4), and fronto-central (FC3/z/4) sites. Mean amplitudes of each component were extracted and, submitted to repeated-measures ANOVA.

Time–frequency analyses were performed in MATLAB using the FieldTrip and WTools toolboxes (Ferrari et al., 2025). For each trial and electrode, we extracted epochs spanning 600 ms before to 1000 ms after target-stimulus onset. Time–frequency decomposition used a fast Fourier transform with a Hanning taper applied to 500-ms windows advanced in 10-ms steps (Handy, 2005). Frequencies from 1 to 30 Hz were analyzed in 1-Hz increments. Oscillatory power was baseline-corrected using the 200-ms pre-target interval and expressed on a decibel scale (dB = 10 × log10 [μV^2^]).To address multiple comparisons, we used FieldTrip’s nonparametric cluster-based permutation approach (Maris & Oostenveld, 2007). Paired-samples t-tests were computed over the three-dimensional time × electrode × frequency space, and adjacent samples (in time, sensor, or frequency) exceeding the cluster-forming threshold (two-tailed, *p* < .025 per tail) were grouped into clusters. For each cluster, the number of suprathreshold samples was used as the cluster-level test statistic (stat_cluster). Regions of interest (ROIs) were then defined based on the topographical distribution of condition differences within the target frequency bands, and 1000 permutation tests were performed at the cluster level. Cluster-level significance was controlled at *p* < .05 (Maris & Oostenveld, 2007; Moreno et al., 2015). Across contexts, condition differences in face recognition were primarily expressed as a significant positive time–frequency cluster over frontal electrodes (F6, F5, F3, F1, Fz, F2, F4) in the theta band (4–8 Hz) from 120 to 310 ms post-stimulus. In contrast, condition differences for self-face recognition manifested as a significant negative time–frequency cluster, comprising alpha-band (8–13 Hz) activity over frontal electrodes (F6, F5, F3, F1, Fz, F2, F4) from 400 to 600 ms, as well as alpha-band activity over centro-parietal electrodes (CPz, CP2, CP4, Pz, P2, P4) from 360 to 580 ms. Greenhouse–Geisser correction was applied to all repeated-measures ANOVAs when necessary, and Bonferroni correction was used for multiple comparisons.

### Context triggered manipulation test

After completing the main task, all participants completed a post-experimental manipulation check for each contextual condition. Three dimensions were assessed: self-relatedness, perceived self threat, and perceived culpability. Ratings were made on 7-point Likert scales ranging from 1 (not at all) to 7 (extremely). These measures were included to clarify the psychological meaning of the contextual manipulation without assuming that crime-related framing necessarily induced subjective self-threat. Self-relatedness ratings were used to verify whether participants experienced the scenario as personally involving. Perceived self threat assessed whether the scenario carried potential defensive significance for the self. Perceived culpability assessed whether participants felt implicated in the wrongdoing described in the scenario.

## Results

### Manipulation check

Manipulation checks were analyzed using one-way repeated-measures ANOVAs with context condition (self-related crime, self-irrelated crime, self-related non-crime) as a within-subject factor. Self-relatedness ratings differed significantly across conditions, *F*(2, 27) = 566.89, *p* < 0.001, *η*_*p*_^*2*^ = .98. Pairwise comparisons showed that the self-related crime condition (*M* = 6.38, *SD* = 0.49) was rated as more self-related than the self-irrelated crime condition (*M* = 1.72, *SD* = 0.59), *t*(28) = 32.61, *p* < .001. The self-related non-crime condition (*M* = 6.24, *SD* = 0.44) was also rated as more self-related than the self-irrelated crime condition, *t*(28) = 32.97, *p* < 0.001. The two self-related conditions did not differ significantly, *p* = 0.31.

Perceived self-image threat also differed across conditions, *F*(2, 27) = 198.44, *p* < 0.001, *η*_*p*_^*2*^ = .94. Ratings were higher in the self-related crime condition (*M* = 5.90, *SD* = 0.82) than in the self-irrelated crime condition (*M* = 3.34, *SD* = 0.81), *t*(28) = 11.62, *p* < 0.001, and the self-related non-crime condition (*M* = 2.03, *SD* = 0.57), *t*(28) = 20.28, *p* < 0.001. The self-irrelated crime condition was also rated as more threatening than the self-related non-crime condition, *t*(28) = 7.03, *p* < 0.001.

Perceived culpability/responsibility showed a significant effect of condition, *F*(2, 27) = 223.93, *p* < 0.001, *η*_*p*_^*2*^ = .94. The self-related crime condition (*M* = 5.34, *SD* = 0.77) was rated higher than both the self-irrelated crime condition (*M* = 1.76, *SD* = 0.51), *t*(28) = 18.97, *p* < 0.001, and the self-related non-crime condition (*M* = 1.52, *SD* = 0.57), *t*(28) = 20.56, *p* < 0.001. The self-irrelated crime and self-related non-crime conditions did not differ significantly, *p* = 0.328.

### Behavioral results

All 29 participants achieved an accuracy rate above 92% in self-face recognition; therefore, all data were included in the analysis. Reaction times (RTs) for incorrect responses and extreme values beyond ± 3 standard deviations in each condition were excluded, resulting in the removal of 4.76% of the data. A 2 (Face Type: self-face vs. other-face) × 3 (Contextual Information: self-related crime vs. self-irrelated crime vs. self-related non-crime) repeated-measures ANOVA was conducted on RTs.

**Fig. 2:**
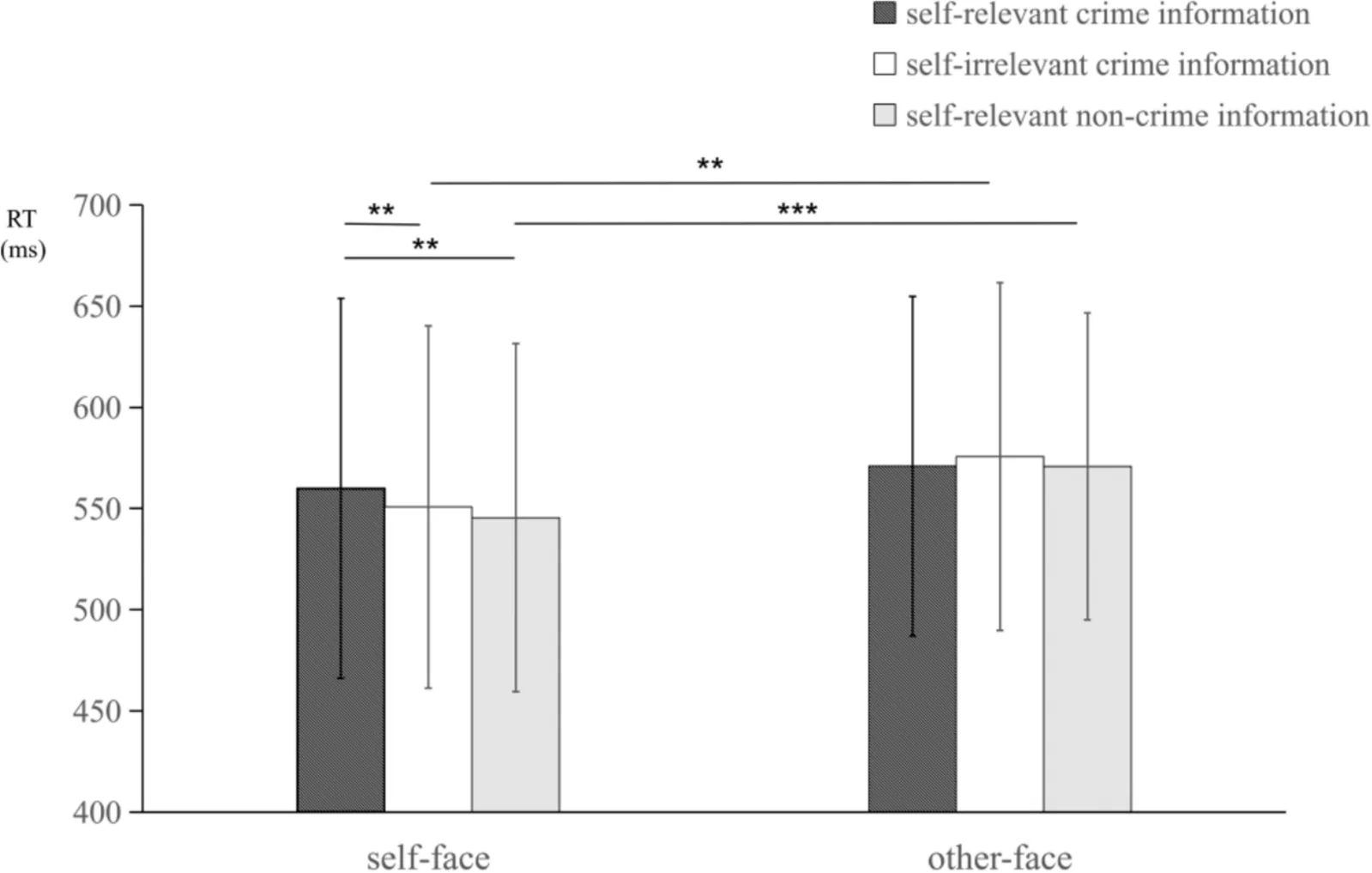
Reaction times in the face recognition task .Error bars represent standard errors, ** *p* < 0.01, ***p < 0.001

The results revealed a significant main effect of face type, *F*(1, 28) = 11.48, *p* < 0.01, *η*_*p*_^*2*^ = 0.29, The main effect of contextual information was not significant, *F*(2, 27) = 2.03, *p* = 0.15. However, the interaction between face type and contextual information was significant, *F*(2, 27) = 3.88, *p* < 0.05, *η*_*p*_^*2*^ = 0.22. Simple effects analysis showed that RTs for self-face recognition differed significantly across contextual conditions, *F*(2, 27) = 4.94, *p* < 0.05, *η*_*p*_^*2*^ = 0.27. Under the self-irrelated crime condition, responses to self-faces were faster than to other-faces, *F*(1, 28) = 13.34, *p* < 0.01, *η*_*p*_^*2*^ = 0.32. Similarly, under the self-related non-crime condition, RTs for self-faces were faster than for other-faces, *F*(1, 28) = 14.93, *p* < 0.001, *η*_*p*_^*2*^ = 0.35. Post-hoc comparisons further indicated that under the self-related crime condition, self-face recognition was slower (*M* = 559.90 ± 17.43 ms; 95% CI [524.20, 595.61]) compared to both the self-related non-crime condition (*M* = 545.48 ± 15.98 ms; 95% CI [512.74, 578.22]), *t*(28) = 3.08, *p* < 0.01, Cohen’s *d* = 1.14, and the self-irrelated crime condition (*M* = 550.75 ± 16.63 ms; 95% CI [516.70, 584.81]), *t*(28) = 3.05, *p* < 0.01, Cohen’s *d* = 1.10. Moreover, self-faces were recognized faster than other-faces in both the self-irrelated crime condition (*M* = 550.75 ± 16.63 vs. 575.69 ± 15.96 ms), *t*(28) = 3.65, *p* < 0.01, Cohen’s *d* = 1.32, and the self-related non-crime condition (*M* = 545.48 ± 15.98 vs. 570.84 ± 14.08 ms), *t*(28) = 3.86, *p* < 0.001, Cohen’s *d* = 1.43 (see Fig. 2).

### Time-domain results

#### N170 (120-180 ms)

**Fig. 3:**
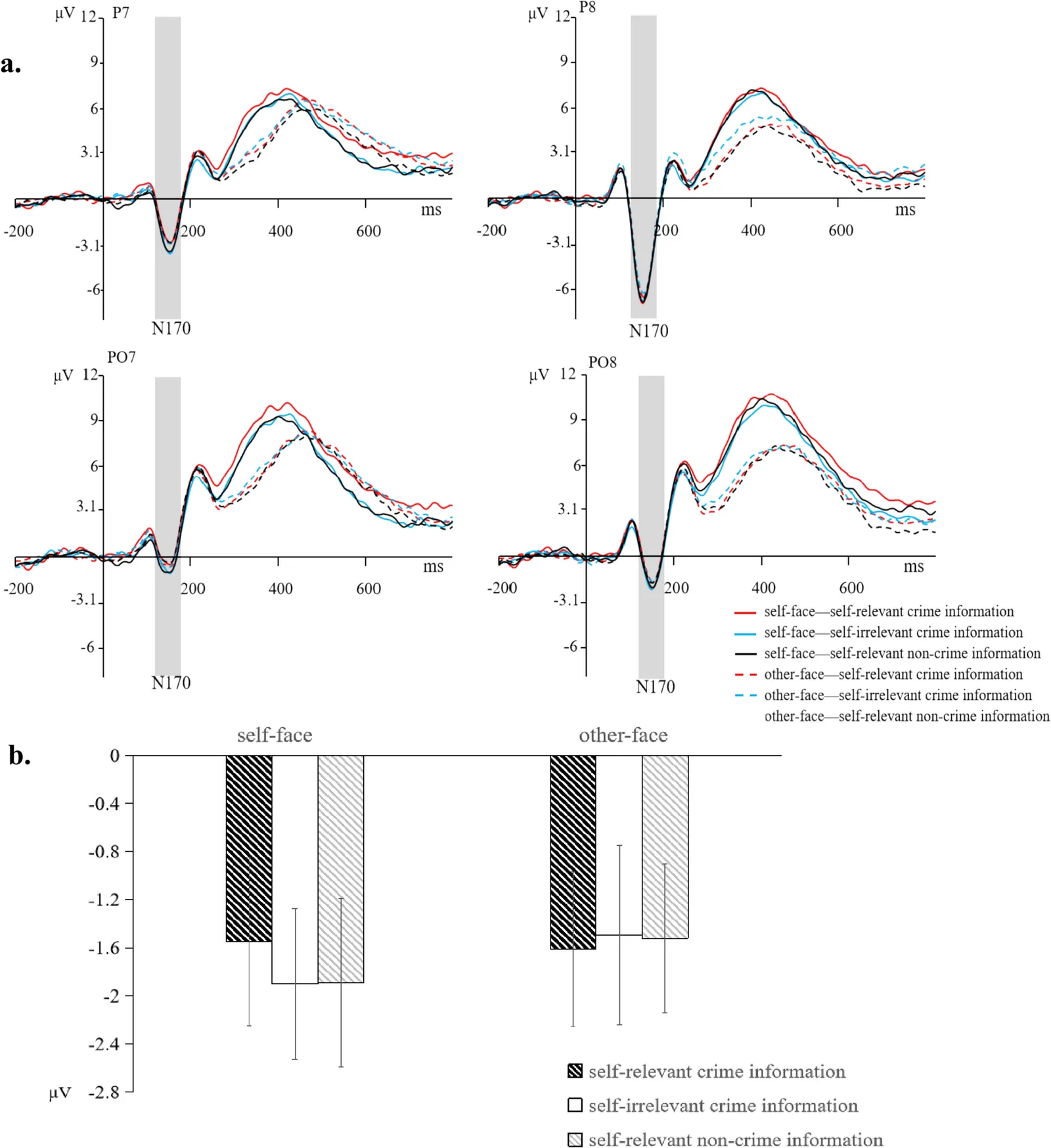
**a** Mean amplitude of N170 evoked by face stimuli in three contextual information conditions; **b** Total average of ERPs evoked on P7, P8, PO7 and PO8 for the two types of faces under the three contextual information. Error bars represent standard errors

A 2 (Face type: self-face vs. other-face) × 3 (Contextual information: self-related crime vs. self-irrelated crime vs. self-related non-crime) repeated-measures ANOVA was conducted on the mean amplitudes of the N170 component. The results revealed that the main effect of face type was not significant, *F*(1, 28) = 2.32, *p* = 0.14; the main effect of contextual information was also not significant, *F*(2, 27) = 0.34, *p* = 0.72; and the interaction between face type and contextual information was not significant, *F*(2, 27) = 1.28, *p* = 0.29 (see Figs. 3 and Fig. 5).

#### N250 (280-340 ms)

A 2 (Face type: self-face vs. other-face) × 3 (Context: self-related crime vs. self-irrelated crime vs. self-related non-crime) repeated-measures ANOVA was conducted on the mean amplitudes of the N250 component. The analysis yielded a significant main effect of face type, *F*(1, 28) = 96.63, *p* < 0.001, *η*_*p*_^*2*^ = 0.78, indicating that self-faces (*M* = 6.71 ± 1.28 μV; 95% CI [4.10, 9.33])elicited significantly larger N250 amplitudes than other-faces (*M* = 2.89 ± 1.12 μV; 95% CI [0.60, 5.17]).

**Fig. 4:**
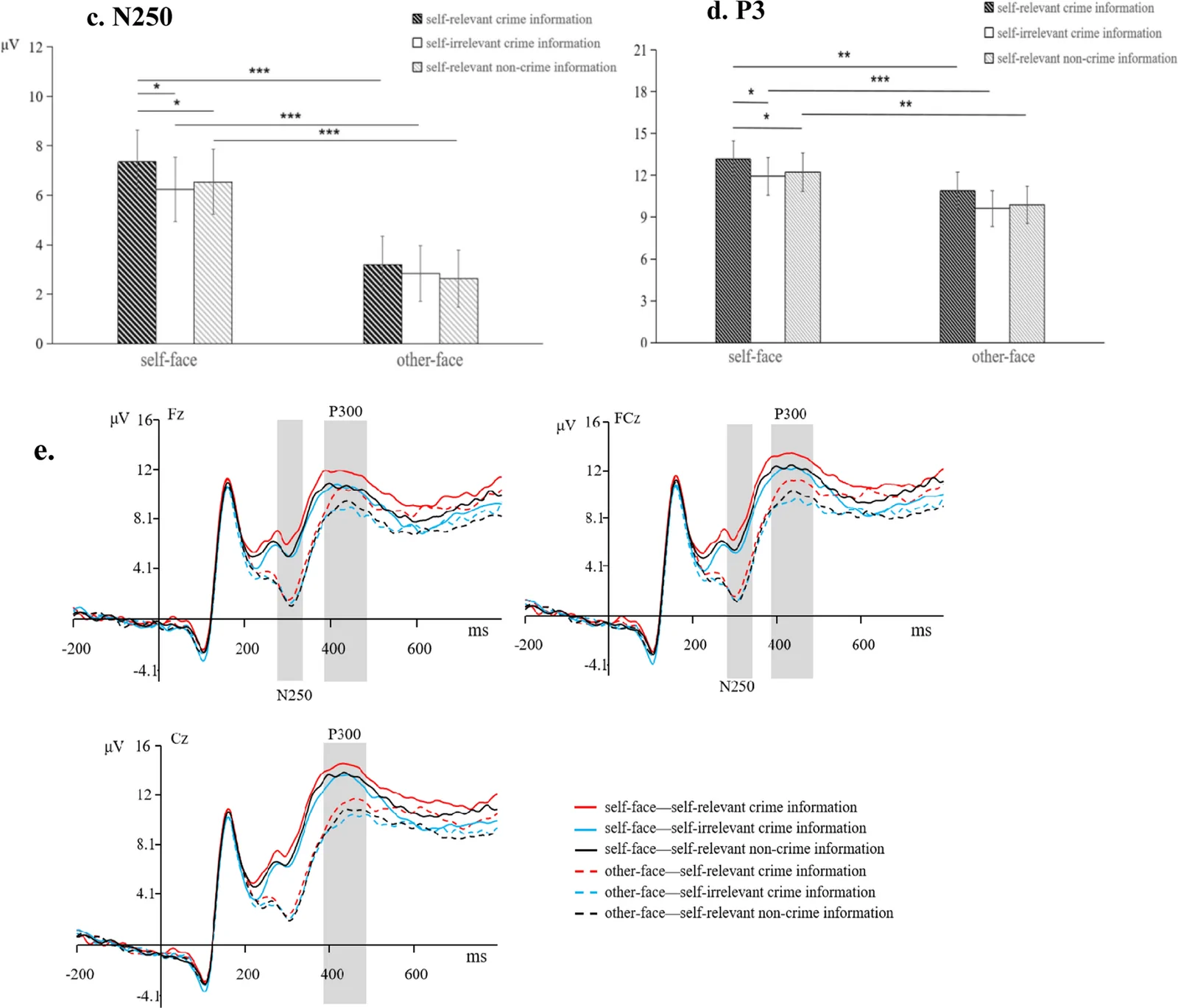
**c**, **d** Mean N250 and P3 amplitudes elicited by self-face and other-face stimuli across the three contextual information conditions; **e**. Total average of ERPs evoked by faces on Fz, FCz, and Cz for the three contextual messages. Error bars represent standard errors, * *p* < 0.05, ** *p* < 0.01, ***p < 0.001

**Fig. 5:**
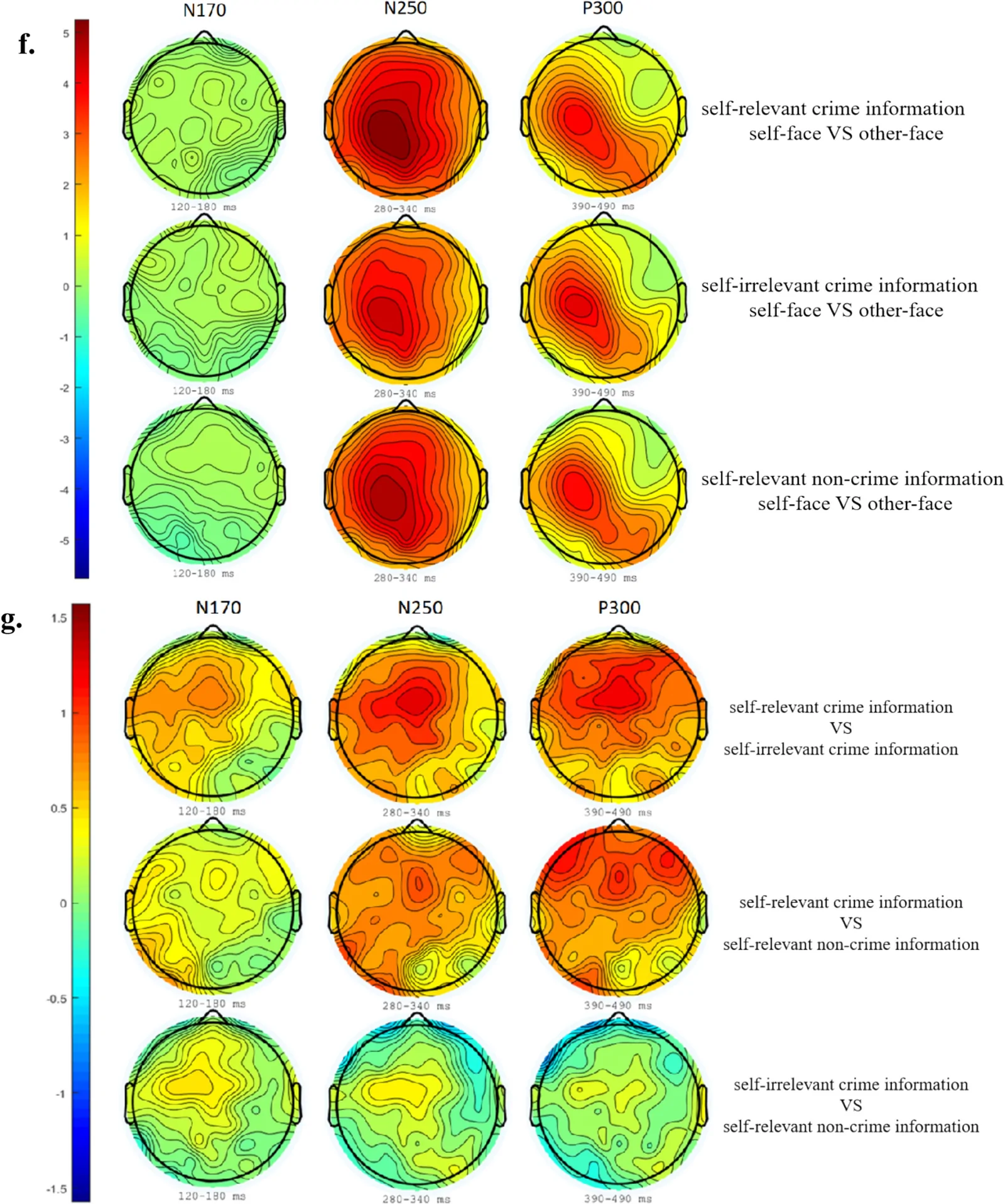
**f**. Differential wave topography of self-face evoked N170, N250, and P3 in three contexts; **g**. Differential wave topography of N170, N250, and P3 evoked by self-face minus other-face in three contexts

The main effect of context was also significant, *F*(2, 27) = 5.14, *p* < 0.05, *η*_*p*_^*2*^ = 0.28. The self-related crime context elicited significantly larger N250 amplitudes overall (*M* = 5.28 ± 1.19 μV; 95% CI [2.83, 7.72]) compared to the self-irrelated crime context (*M* = 4.53 ± 1.20 μV; 95% CI [2.09, 6.98]) and the self-related non-crime context (*M* = 4.59 ± 1.19 μV; 95% CI [2.15, 7.03]). The difference between the self-irrelated crime and self-related non-crime contexts was not significant (*p* = 1.000). And the Face type × Context interaction was not significant, *F*(2, 27) = 1.15, *p* = 0.33 (see Figs. 4 and 5).

#### P3 (390-490 ms)

A 2 (face type: self-face vs. other-face) × 3 (contextual information: self-related crime, self-unrelated crime, self-related non-crime) repeated-measures ANOVA was conducted on the mean amplitudes of the P3 component. The results revealed a significant main effect of face type, *F*(1, 28) = 16.89, *p* < 0.001, *η*_*p*_^*2*^ = 0.38. Across contextual conditions, self-faces (*M* = 12.44 ± 1.32 μV; 95% CI [9.74, 15.14]) elicited larger P3 amplitudes than other-faces (*M* = 10.13 ± 1.30 μV; 95% CI [7.46, 12.79]).

The main effect of contextual information was also significant, *F *(2, 27) = 8.03, *p* < 0.01, *η*_*p*_^*2*^ = 0.37. The self-related crime condition (*M* = 12.01 ± 1.30 μV; 95% CI [9.35, 14.68]) elicited larger P3 amplitudes than both the self-unrelated crime condition (*M* = 10.77 ± 1.28 μV; 95% CI [8.15, 13.40]) and the self-related non-crime condition (*M* = 11.06 ± 1.30 μV; 95% CI [8.41, 13.71]), both ps < .05. The difference between the self-unrelated crime and self-related non-crime conditions was not significant, *p* = 1.00. The interaction between face type and contextual information was not significant, *F* (2, 27) = 0.02, *p* = 0.98 (see Figs. 4 and 5).

### Time frequency results

#### Theta (4–8 Hz)

**Fig. 6:**
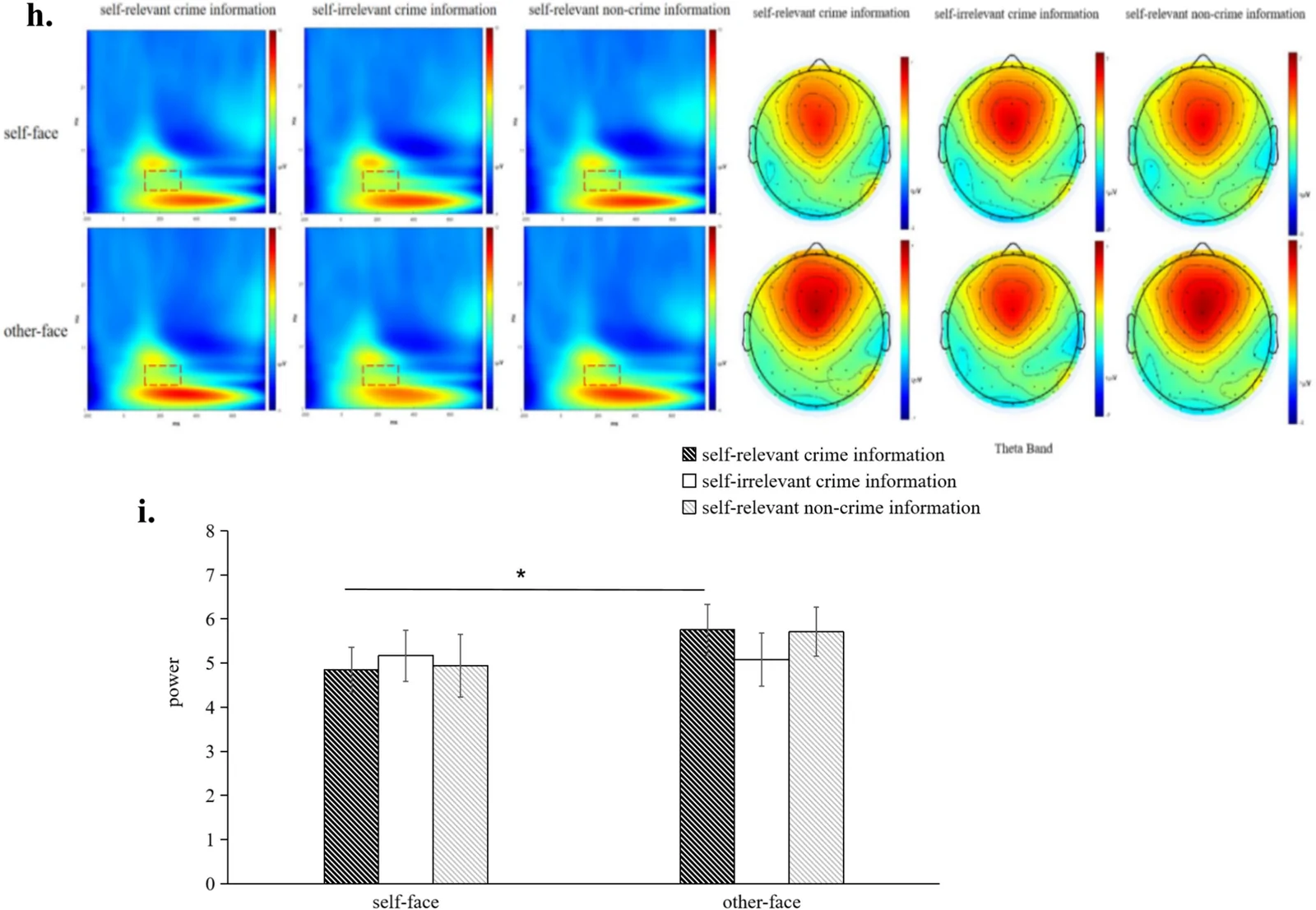
**h**. Time–frequency plots of Theta oscillations in the frontal region (F6, F5, F3, F1, Fz, F2, F4) for three contextually informative face recognition, rectangles denote the time windows used to analyze the Theta oscillations; **i**. Theta energy in frontal regions. Error bars represent standard errors represents, *p < 0.05

A 2 (face type: self-face vs. other-face) × 3 (contextual information: self-related crime vs. self-irrelated crime vs. self-related non-crime) repeated-measures ANOVA was conducted on the mean Theta power at frontal electrodes. The results revealed a significant main effect of face type, *F*(1, 28) = 4.68, *p* < 0.05, *η*_*p*_^*2*^ = 0.14. In the self-related crime condition, self-faces (*M* = 4.85 ± 0.51 dB; 95% CI [3.80, 5.89]) elicited significantly lower Theta power compared to other-faces (*M* = 5.75 ± 0.58 dB; 95% CI [4.56, 6.95]), *t*(28) = 2.18, *p* < 0.05, Cohen’s *d* = 0.81.The main effect of contextual information was not significant, *F*(2, 27) = 0.38, *p* = 0.69, and the interaction between face type and contextual information was also not significant, *F*(2, 27) = 1.73, *p* = 0.19 (see Fig. 6).

#### Alpha (8–13 Hz)

A 2 (face type: self-face vs. other-face) × 3 (contextual information: self-related crime, self-unrelated crime, self-related non-crime) repeated-measures ANOVA was conducted on the mean Alpha power at frontal electrodes. The main effect of contextual information was not significant, *F*(2, 27) = 1.71, *p* = 0.19, and the main effect of face type was also not significant, *F*(1, 28) = 0.35, *p* = 0.56. However, the interaction between contextual information and face type was significant, *F*(2, 27) = 4.50, *p* < 0.05, *η*_*p*_^*2*^ = 0.25. To clarify this interaction, follow-up analyses examined the simple effect of face type within each contextual condition. Bonferroni-corrected pairwise comparisons were used. Alpha power differed significantly between self- and other-face recognition only in the self-related non-crime condition, *F*(1, 28) = 6.97, *p* < 0.05, *η*_*p*_^*2*^ = 0.20. Specifically, self-face recognition showed more negative Alpha power (*M* = –1.30 ± 0.74 dB; 95% CI [–2.81, 0.20]) than other-face recognition (*M* = –0.61 ± 0.65 dB; 95% CI [–1.93, 0.72]), *t*(28) = 2.64, *p* < 0.05, Cohen’s *d* = 0.98.

**Fig. 7:**
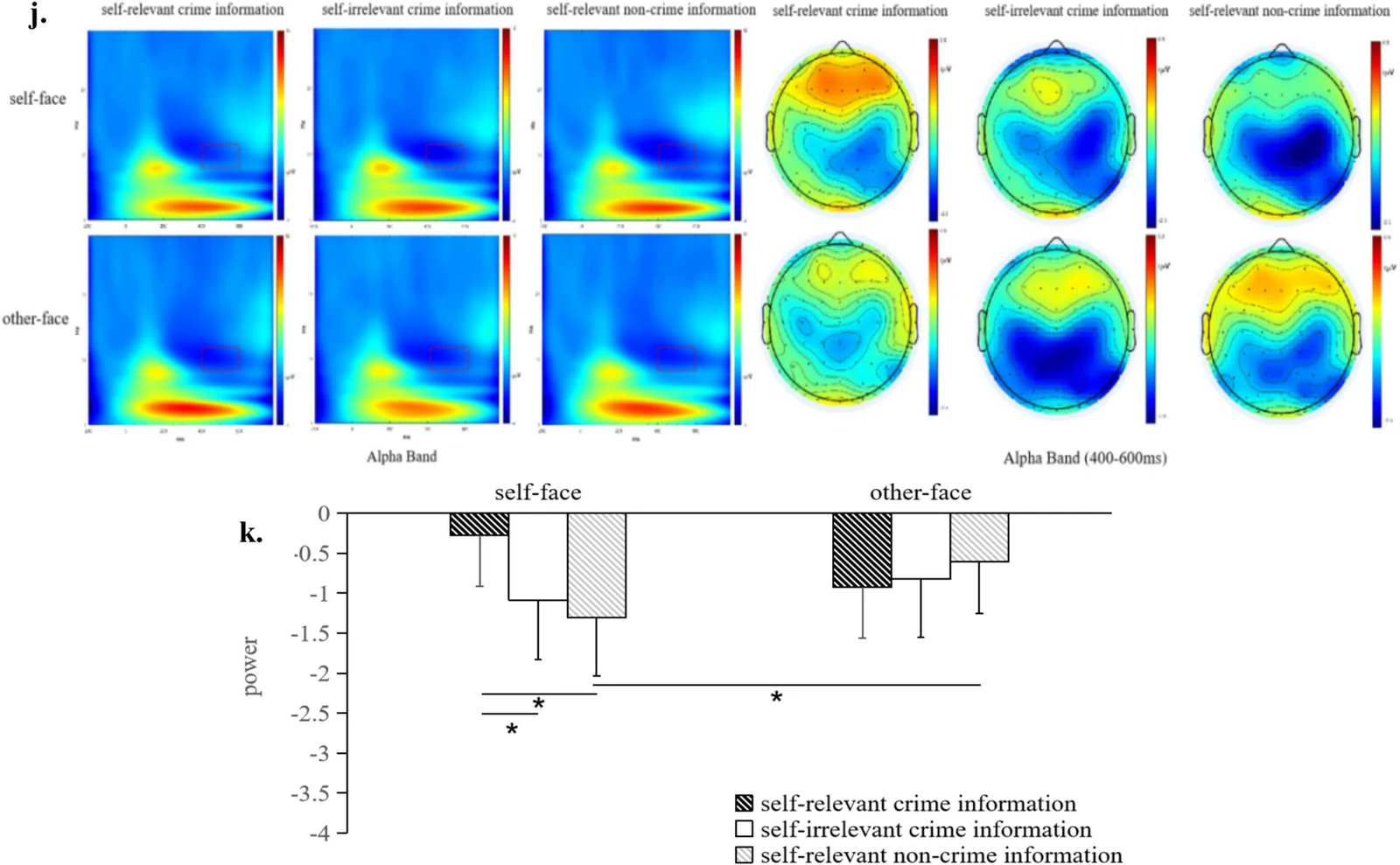
**j**. Time–frequency plots of alpha oscillations in the frontal region (F6, F5, F3, F1, Fz, F2, F4) for the three contextually informative face recognitions, with the rectangles at denoting the time windows used to analyze the alpha oscillations; **k**. Alpha energy of frontal regions. Error lines represent standard errors, * *p* < 0.05

Because the present study focused on how contextual information modulates self-face processing, we further examined contextual differences within self-face trials. Alpha power for self-face recognition differed significantly across contextual conditions, *F*(2, 27) = 3.96, *p* < 0.05, *η*_*p*_^*2*^ = 0.23. Bonferroni-corrected comparisons showed that Alpha power was less negative in the self-related crime condition (*M* = –0.28 ± 0.64 dB; 95% CI [–1.58, 1.02]) than in the self-unrelated crime condition (*M* = –1.09 ± 0.74 dB; 95% CI [–2.61, 0.43]), *t*(28) = 2.62, *p* < 0.05, Cohen’s *d* = 0.97, and the self-related non-crime condition (*M* = –1.30 ± 0.73 dB; 95% CI [–2.81, 0.20]), *t*(28) = 2.66, *p* < 0.05, Cohen’s *d* = 0.99 (see Fig. 7).

**Fig.8:**
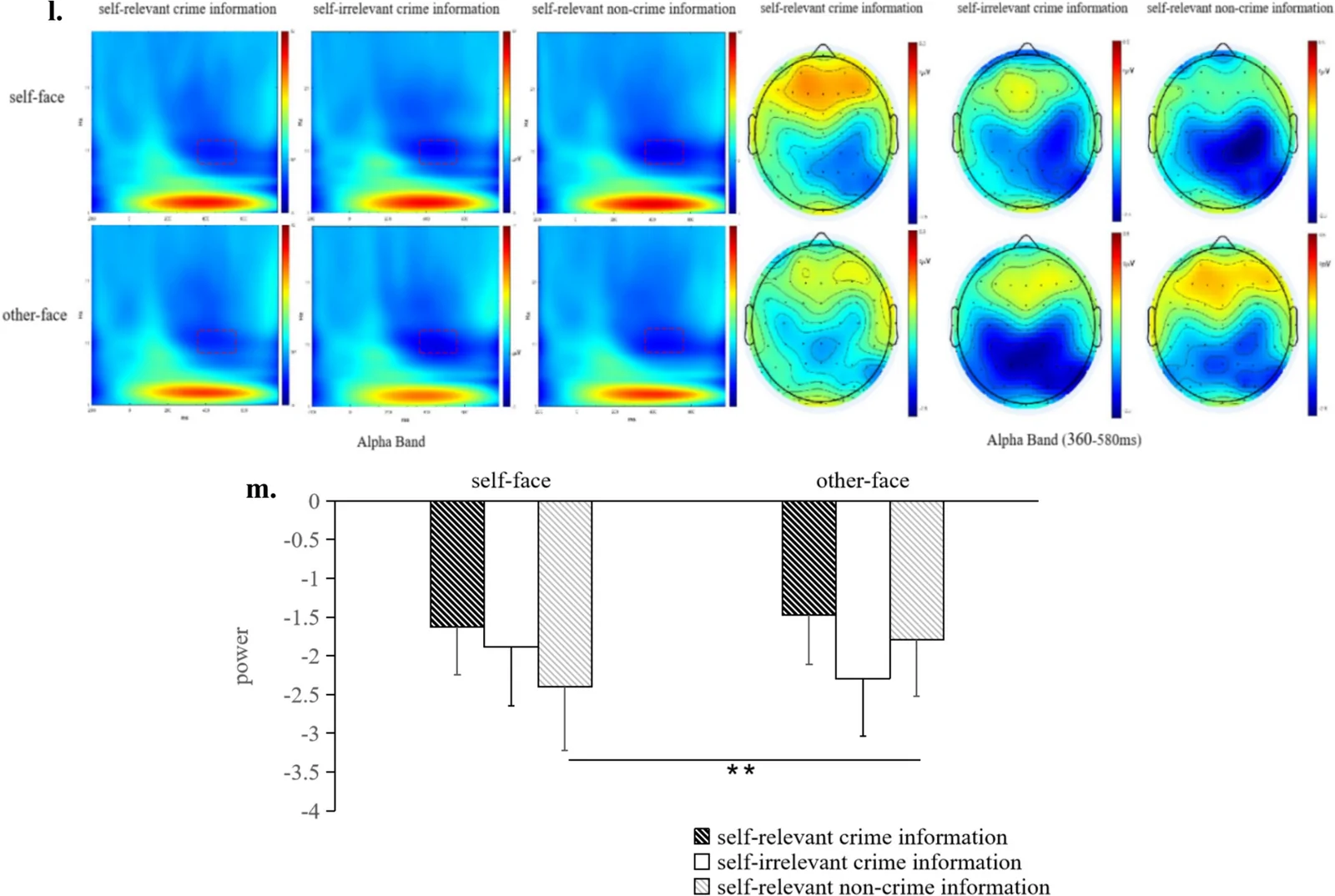
**l**. Time–frequency plots of alpha oscillations in the central-parietal region (CPz, CP2, CP4, Pz, P2, P4) for the three contextual information face recognition. Rectangles indicate the time windows used to analyze the alpha oscillations. m. Alpha energy of the central-parietal region error lines represent standard errors, ** *p* < 0.01

A 2 (face type: self-face vs. other-face) × 3 (contextual information: self-related crime, self-unrelated crime, self-related non-crime) repeated-measures ANOVA was also conducted on the mean Alpha power at centro-parietal electrodes. The main effect of contextual information was not significant, *F*(2, 27) = 2.85, *p* = 0.07, and the main effect of face type was not significant, *F*(1, 28) = 0.74, *p* = 0.40. However, the interaction between contextual information and face type was significant, *F*(2, 27) = 3.78, *p* < 0.05, *η*_*p*_^*2*^ = 0.22. Follow-up analyses again examined the simple effect of face type within each contextual condition, with Bonferroni correction applied to pairwise comparisons. Alpha power differed significantly between self- and other-face recognition only in the self-related non-crime condition, *F*(1, 28) = 7.82, *p* < 0.01, *η*_*p*_^*2*^ = 0.22. Specifically, self-face recognition showed more negative Alpha power (*M* = –2.40 ± 0.82 dB; 95% CI [–4.08, –0.71]) than other-face recognition (*M* = –1.80 ± 0.73 dB; 95% CI [–3.29, –0.30]), *t*(28) = 2.80, *p* < 0.01, Cohen’s *d* = 1.03 (see Figs. 8).

## Discussion

This study examined how self-face processing is shaped by crime-related contextual framing. Self-related crime framing does not suppress self-processing. Instead, it preserves core self-prioritization while altering the efficiency and control dynamics through which self-relevant information is translated into behavior. Behaviorally, self-faces retained an overall advantage over other-faces, although responses to self-faces were slower in the self-related crime condition. In the ERP domain, self-faces elicited larger N250 and P3 amplitudes across contexts, indicating preserved identity activation and post-perceptual evaluation. Meanwhile, crime-related framing produced broader N250 and P3 effects, suggesting increased general salience or evaluative significance during face processing. Theta and alpha activity further showed context-dependent modulation. This pattern suggests that self-implicating negative contexts may recalibrate the control and attentional dynamics through which self-related information guides behavior. The manipulation check supports this interpretation: the self-related crime condition was rated as more self-related, self-threatening, and culpability-related than the other conditions, indicating that participants differentiated it from both self-irrelated crime and self-related non-crime contexts.

### Self-prioritization is preserved but behaviorally recalibrated by crime context

The present findings indicate relative representational stability alongside context-dependent changes in processing efficiency. Across conditions, self-faces were judged faster and elicited larger N250 and P3 amplitudes than other-faces, consistent with enduring self-prioritization and with the Self-Attention Network account of prioritized engagement with self-relevant cues (Humphreys & Sui, 2016; Sui & Rotshtein, 2019). Importantly, this effect remained detectable even in crime-related contexts, suggesting that core self-representations were relatively robust rather than easily suppressed by contextual framing. At the same time, self-related crime framing altered the efficiency with which self-related information was translated into responses. Responses to self-faces were slower in this condition, indicating that the behavioral self-advantage was attenuated but not eliminated. This slowing co-occurred with theta and alpha modulation, which may reflect altered control dynamics, heightened vigilance, or changes in attentional gating. A defensive-processing account is compatible with this pattern, especially given the manipulation-check results, but it remains one plausible interpretation rather than a definitive mechanism.

Contextual ERP effects were not self-specific. The self-related crime condition increased N250 and P3 amplitudes for both self- and other-faces, with no reliable face type × context interaction. This suggests that crime-related framing may have acted as a globally salient contextual prime, enhancing identity-related and evaluative processing for socially relevant faces more broadly rather than selectively weakening self-signals. Thus, crime-related contexts did not abolish self-prioritization; instead, they reshaped its behavioral expression while leaving core neural indices of self-processing relatively stable.

Importantly, the behavioral slowing in the self-related crime condition should not be interpreted as a loss of self-prioritization. The ERP results showed that self-faces continued to elicit enhanced N250 and P3 responses across contexts, suggesting that self-related identity activation and post-perceptual evaluation remained robust. Thus, the self-related crime context appears to have affected the efficiency with which self-related representations were translated into overt responses, rather than weakening the underlying self-representation itself.

### Effects of contextual information on self-face processing

#### Early processing: stable stru ctural encoding and altered control dynamics

At the earliest perceptual stage, N170 was not modulated by face type or context, consistent with its role in structural face encoding and its relative insensitivity to higher-order factors such as identity and contextual meaning (Eimer, 2012; Gao et al., 2025; Olivares et al., 2015). Thus, early structural encoding appeared comparatively stable even when faces were preceded by crime-related contextual information. Oscillatory activity, however, suggested that contextual information may have influenced processing strategy. The reduction in frontal theta in the self-related crime condition diverges from the classic pattern in which increased midfrontal theta reflects conflict monitoring and control engagement (Cavanagh & Frank, 2014). One possible interpretation is that reduced theta reflects a shift in control strategy, such as reduced elaborative engagement, altered monitoring, or inhibitory gating when crime-related meaning is linked to the self (Codispoti et al., 2023; Wang et al., 2022). Nevertheless, theta activity is functionally heterogeneous. This effect is therefore best interpreted as altered control dynamics rather than direct evidence of defensive disengagement or self-protective inhibition.

The absence of N170 modulation suggests that contextual framing did not substantially alter the early structural encoding of faces. This finding is theoretically important because it indicates that the effects of self-related crime framing emerged primarily after initial perceptual analysis. The theta result should be interpreted more cautiously. Although frontal theta is often associated with conflict monitoring and cognitive control, the present theta effect did not provide decisive evidence for a specific defensive mechanism. Rather, it suggests that self-related crime framing may have altered the control state under which self-face processing occurred, possibly involving changes in monitoring, gating, or task engagement.

#### Mid-to-late processing reflects preserved self-advantage and generalized contextual salience

At the mid-stage indexed by N250, self-faces showed a consistent advantage across contexts, supporting robust and relatively automatic self-identity activation (Sama et al., 2024; Schweinberger & Neumann, 2016). The self-related crime condition increased N250 amplitudes for both self- and other-faces, suggesting that crime-related framing enhanced identity-related processing broadly rather than selectively disrupting self-face processing. P3 results showed a similar pattern. Self-faces continued to elicit larger P3 amplitudes than other-faces, consistent with preferential post-perceptual resource allocation to self-relevant stimuli (Cygan et al., 2014; Sui & Gu, 2017). At the same time, self-related crime increased P3 amplitudes overall, suggesting that crime-related contextual information increased the evaluative significance or processing demands of face stimuli in general (Schellhaas et al., 2022). These results are therefore more consistent with preserved self-prioritization plus generalized contextual enhancement than with selective suppression of self-processing.

The N250 and P3 findings point to two separable effects. First, self-faces elicited larger amplitudes than other-faces, supporting preserved self-related identity activation and preferential post-perceptual evaluation. Second, the self-related crime context increased N250 and P3 amplitudes more broadly. Because the face type × context interactions were not significant, these ERP effects should not be interpreted as selective enhancement of self-face processing under crime-related framing.

In contrast to the N250 and P3 effects, alpha-band activity showed a clearer face type × context interaction. This suggests that alpha activity may be more sensitive to the conjunction of self-relevance and crime-related meaning than canonical ERP amplitudes. The direction of the frontal alpha effect should be described in terms of the magnitude of the negative-going alpha response: for self-face recognition, self-related crime information was associated with a smaller alpha-response amplitude than the other contexts. One interpretation is that this reduced alpha response reflects altered attentional gating, a more conservative control state, or changes in top-down regulation when self-identity is paired with self-related crime information.

#### A recalibration account of self-prioritization in socially evaluative contexts

Together, the findings support a dual-level account of crime-related contextual modulation. At the representational level, self-prioritization remained robust: self-faces showed an overall behavioral advantage and elicited larger N250 and P3 amplitudes than other-faces across contexts. This indicates that self-related information retained privileged access to identity activation and post-perceptual evaluation, consistent with the Self-Attention Network account (Humphreys & Sui, 2016; Sui & Rotshtein, 2019). The results are also compatible with the Implicit Positive Association perspective, insofar as self-relevant signals remained potent even when their contextual meaning was negative and self-implicating (Ma & Han, 2010).

At the same time, behavioral and oscillatory findings suggest context-dependent reconfiguration of processing efficiency and control dynamics. Slower responses to self-faces in the self-related crime condition indicate that the self-advantage was attenuated in behavioral expression, despite preserved ERP indices of self-processing. Reduced frontal theta and the reduced magnitude of the negative-going frontal alpha response for self-faces under self-related crime framing further suggest changes in attentional gating or control configuration. One possible interpretation is that self-related crime framing increased defensive significance, leading to a more regulated or conservative processing mode.

Thus, the present data do not suggest that crime-related contexts suppress or eliminate self-processing. Rather, self-processing remained relatively stable at core representational stages, while its behavioral efficiency and oscillatory control dynamics were modulated by contextual meaning. Crime-related framing therefore appears to recalibrate the expression of self-prioritization rather than undermine the self-prioritization effect itself.

### Contribution and limitation

A central theoretical contribution of the present study is that frontal alpha oscillations may represent a key locus where self-relevance and self-implicating crime-related context jointly modulate self-face processing. Whereas conventional ERP markers (e.g., N250 and P3) were consistent with a robust self-prioritization effect that appeared relatively context-general, the frontal alpha interaction suggested a more selective reconfiguration: self-related crime cues were associated with a smaller negative-going frontal alpha response during self-face recognition than the other contexts. This pattern is compatible with a layered control architecture in which stable identity/semantic processing is preserved, yet the expression of self-related representations is flexibly shaped by context-sensitive frontal gating. In this view, self-prioritization need not reflect uniform attentional amplification; instead, it may involve context-dependent monitoring, gating, or regulation specifically targeted at self-cues under self-implicating threat. These findings may help refine both the Self-Attention Network and Implicit Positive Association accounts by providing convergent evidence that self-representations can remain robust when positive self-associations are challenged, while regulatory configuration—indexed here by frontal alpha—varies with self-threatening context.

Despite its contributions, the present study has several limitations that warrant further refinement. First, the contextual conditions may have differed not only in self-relevance and crime-related meaning, but also in emotional salience, perceived severity, plausibility, and arousal. Although the design attempted to separate self-relevance from crime relevance, future studies should more tightly match contextual materials on valence, arousal, familiarity, semantic complexity, and perceived plausibility. This would help determine whether the observed effects are driven specifically by self-related crime framing or by broader contextual salience. Second, in this study, alpha oscillations were interpreted as reflecting a context-dependent change in attentional gating or control configuration. However, the functional role of alpha activity may be bidirectional, involving both task inhibition and perceptual facilitation. Future research should incorporate manipulations such as cue validity or task difficulty to systematically test the functional relationship between alpha activity and processing efficiency.

## Conclusion

In conclusion, self-related crime framing did not eliminate the self-face advantage. Instead, self-prioritization was preserved at the level of identity activation and post-perceptual evaluation, while its behavioral expression and oscillatory control dynamics were recalibrated by contextual meaning. These findings suggest that self-processing is both robust and flexible: self-relevant information retains privileged processing status, but the cognitive system adjusts how such information is controlled and translated into action when it is embedded in self-related crime contexts.
